# Tiered approach for the identification of Mal d 1 reduced, well tolerated apple genotypes

**DOI:** 10.1038/s41598-020-66051-4

**Published:** 2020-06-04

**Authors:** Emilia Romer, Soraya Chebib, Karl-Christian Bergmann, Katrin Plate, Sylvia Becker, Christina Ludwig, Chen Meng, Thilo Fischer, Werner Dierend, Wilfried Schwab

**Affiliations:** 1 Biotechnology of Natural Products, Technical University Munich, Liesel-Beckmann-Str. 1, 85354 Freising, Germany; 2 Comprehensive Allergy Center Charité, Charité Hospital Berlin, Charitéplatz 1, 10117 Berlin, Germany; 3 Faculty of Agricultural Science and Landscape Architecture, Fruit Science, University of Applied Sciences Osnabrück, Oldenburger Landstr. 24, 49090 Osnabrück, Germany; 4 European Center of Allergy Research Foundation, Robert-Koch-Platz 7, 10117 Berlin, Germany; 5 Bavarian Center for Biomolecular Mass Spectrometry (BayBioMS), Technical University Munich, Gregor-Mendel-Straße 4, 85354 Freising, Germany; 6 4GENE, Lise-Meitner-Str. 30, 85354 Freising, Germany

**Keywords:** Proteins, Proteomics, Nutrition

## Abstract

A rising proportion of the world population suffers from food-related allergies, including incompatibilities to apples. Although several allergenic proteins have been found in apples, the most important proteins that cause allergic reactions to apples in Central-Northern Europe, and North America are the Mal d 1 proteins, which are homologues of the birch pollen allergen Bet v 1. As the demand for hypoallergenic fruits is constantly increasing, we selected apple genotypes with a low total content of Mal d 1 by enzyme-linked immunosorbent assay analysis from segregating populations and tested the tolerability of these fruits through a human provocation study. This tiered approach, which exploited the natural diversity of apples, led to the identification of fruits, which were tolerated by allergic patients. In addition, we found a significant correlation (coefficient >0.76) between the total Mal d 1 content and flavan-3-ol amount and show that the isoform composition of the Mal d 1 proteins, which was determined by LC-MS/MS has a decisive effect on the tolerability of apple genotypes. The approach presented can be applied to other types of fruit and to other allergenic proteins. Therefore, the strategy can be used to reduce the allergen content of other plant foods, thereby improving food safety for allergy subjects.

## Introduction

The apple fruit (*Malus* × *domestica* Borkh.) from the family of Rosaceae is cultivated and consumed worldwide. With a production of over 83 million tons in 2017, apple is one of the most economically important fruits in the world^1^. However, for more and more people, eating apples is becoming increasingly unpleasant and sometimes dangerous due to an allergic reaction (called oral allergy syndrome) against certain apple proteins of the pathogen-related protein family (PR-proteins). PR-proteins can be classified into 17 different families according to their different functions in coping with abiotic and biotic stress conditions and associated defence mechanisms of plants, such as antifungal activity, RNAse activity, or their involvement in the transport of hormones and fatty acids^2–4^. Some PR-proteins have the ability to trigger a variety of allergies, including hay fever and plant-based food allergies^2,5^. The occurrence of different types of apple allergies and their causative proteins depends on the geographical location. In Mediterranean regions Mal d 3, a lipid-transfer-protein (LTP) of the PR-14 protein family is the major apple allergen, while most people in the Northern and Middle parts of Europe and North America suffer from an allergy against Mal d 1. Mal d 1 is a homologous protein to Bet v 1, the major birch pollen allergen and belongs the PR-10 protein family^5^. This protein is present in the flesh and the skin of the fruits^5^, as well as in leaves^6^, and pollen of the trees^7^.

In Europe, 8% to 16% of the population show an allergic reaction to birch pollen^8^. The proportion of people suffering from cross-reactivity between birch pollen allergy and apple allergy is between 47%^9^ and 80%^10^ and is increasing during the last years. The origin of this increase remains unknown^11^. Therefore, the need for breeding hypoallergenic fruits is constantly increasing. Although RNAi successfully reduced Mal d 1 expression in *in vitro*-grown apple plantlets as reported in 2005 and the research few years later lead to genetically modified hypoallergenic apple varieties^6,12,13^, this strategy has no future in Europe because the use of genetically modified food is not permitted. However, the allergenicity of apples and their tolerance in apple-allergic subjects is cultivar dependent, affected by cultivation conditions and therefore, should allow the selection of hypoallergenic genotypes^14^.

Mal d 1 is encoded by more than 30 different genes, which result in a high diversity of different protein isoforms^15^. It is assumed that these different isoallergens show a varying allergenic potential but the exact context is still unknown. The gene expression of the different isoallergens has been extensively studied^12,15,16^. ELISA testing, routinely used to determine Mal d 1 in apples detects different types of isoforms depending on the antibody used^5,14,17,18^. However, until now, no proteomic analysis has been accomplished to analyse the protein content of the different Mal d 1 isoforms.

In addition to the PR-proteins, plants have various techniques to cope with abiotic and biotic stresses. The production of polyphenols and polyphenol-glycosides is an important defence mechanism of plants^19,20^. Polyphenols are secondary metabolites with multiple functions in plants^21^, such as protection against UV radiation^22^ and pathogens like fungi^23^ and insects^24^. These substances benefit human health by reducing the risk of cardiovascular diseases, blood pressure and cancer^25,26^. In apples, the main polyphenols are flavan-3-ols, flavonols, phenolic acids, mainly chlorogenic acid, anthocyanins and exclusively in apples, the dihydrochalcones phloretin and phloridizin^27,28^. Like the concentration of allergy-inducing PR-proteins, the content of the polyphenols in apple is cultivar dependent. Some old apple varieties show a higher amount of polyphenols than new cultivars like Golden Delicious or Cripps Pink (Pink Lady)^29^. Previous studies also indicated a correlation between allergenicity and total polyphenol content^30,31^. Apples with a higher polyphenol content seem to be better tolerated by individuals affected by an allergy^32^.

The demand for hypoallergenic apples is constantly increasing. Since a GMO-approach has so far not led to the desired results, we aimed to develop a non-GMO strategy to identify hypoallergenic apple genotypes in breeding lines taking advantage of the natural genetic diversity of the Mal d 1 content. We identified progenies with reduced Mal d 1 concentration by ELISA testing and confirmed the tolerability by human studies in oral provocation tests. The quantification of polyphenols and proteome analysis provided new insights into the roles of the natural products and isoallergens for the onset of allergy. This information serves as a basis for the breeding of hypoallergenic apples.

## Results

### Mal d 1 content in apples of breeding lines determined by ELISA-assay

Indirect competitive ELISA-assays were performed with a polyclonal antibody raised against the recombinant Mal d 1 protein to detect as many Mal d 1 isoforms as possible. A standard curve with a linear range between 0.1 and 10 µg/ml was measured on each plate. The Mal d 1 content of more than 100 progenies of cross-breeding populations of 2016, 2017 and 2018 (Supplementary material S1), which were already preselected according to yield, fruit size, disease susceptibility, appearance and taste, were compared with the allergen levels of three established cultivars, Golden Delicious, Santana and Braeburn. Golden Delicious is a cultivar known for its high allergenic potential^5^, Braeburn shows a medium allergenicity^30,33^, while Santana is a cultivar that is mostly well tolerated by allergic patients^34^. After each year, genotypes with unsuitable characteristics were removed from the programme and new genotypes were added for the following year. Therefore, 3-year data are not available for comparison for all genotypes.

**Figure 1:**
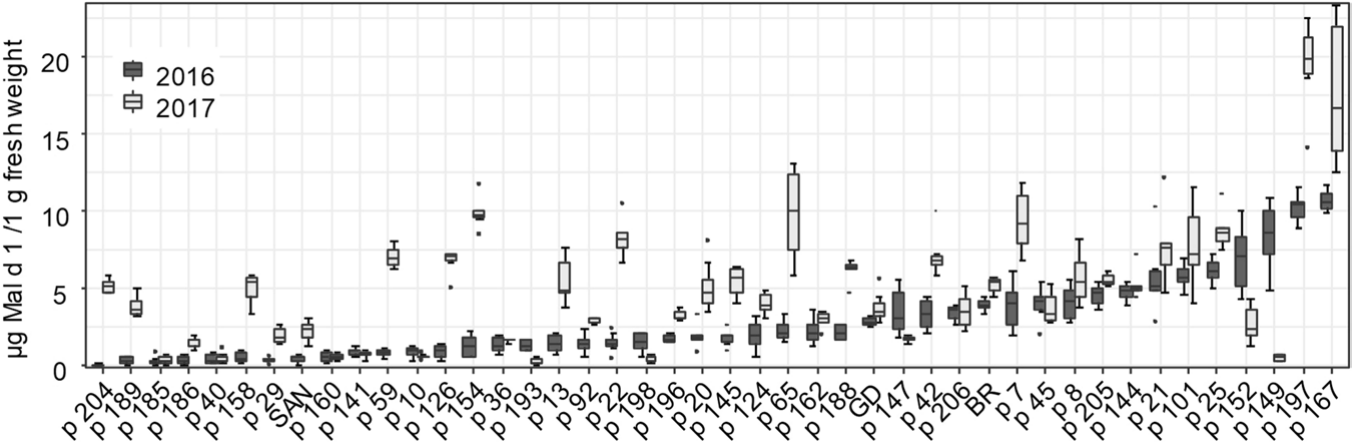
Mal d 1 content in µg/g fresh weight of apple genotypes of the cross breeding population and three standard cultivars (Santana (SAN), Golden Delicious (GD) and Braeburn (BR) determined with ELISA-assay of two different harvest years 2016 (black) and 2017 (grey). The image was generated with RStudio 3.6.0 (https://rstudio.com).

The Mal d 1 concentrations of selected genotypes of the segregating populations varied between 0.07 µg/g fresh weight (fw) of genotype p204 of 2016 and 20.4 µg/g fw of p197 of 2017 with most progenies containing less than 10 µg Mal d 1 /g fw (Fig. 1; Supplementary material S1). The hypoallergenic Santana apples contained 0.5 µg/ Mal d 1 /g fw in 2016 and 2.3 µg Mal d 1 /g fw in 2017. Golden Delicious showed only a slightly higher Mal d 1 concentration compared with Santana (2.9 µg/g fw in 2016 and 3.4 µg/g fw in 2017), even though it is known for its high allergenic potential.

For some genotypes, the allergen content varied strongly between the different cultivation years (Fig. 1). This is exemplified by the genotypes p158, p59, p126, p154, p13, p22 and p65, which showed a much higher Mal d 1 concentration in 2017. In contrast, the allergen content of the genotypes p147, p152 and p149 was lower in 2017 than in the previous year. Other genotypes showed similar Mal d 1 levels in both years.

### Human study with provocation tests

**Figure 2:**
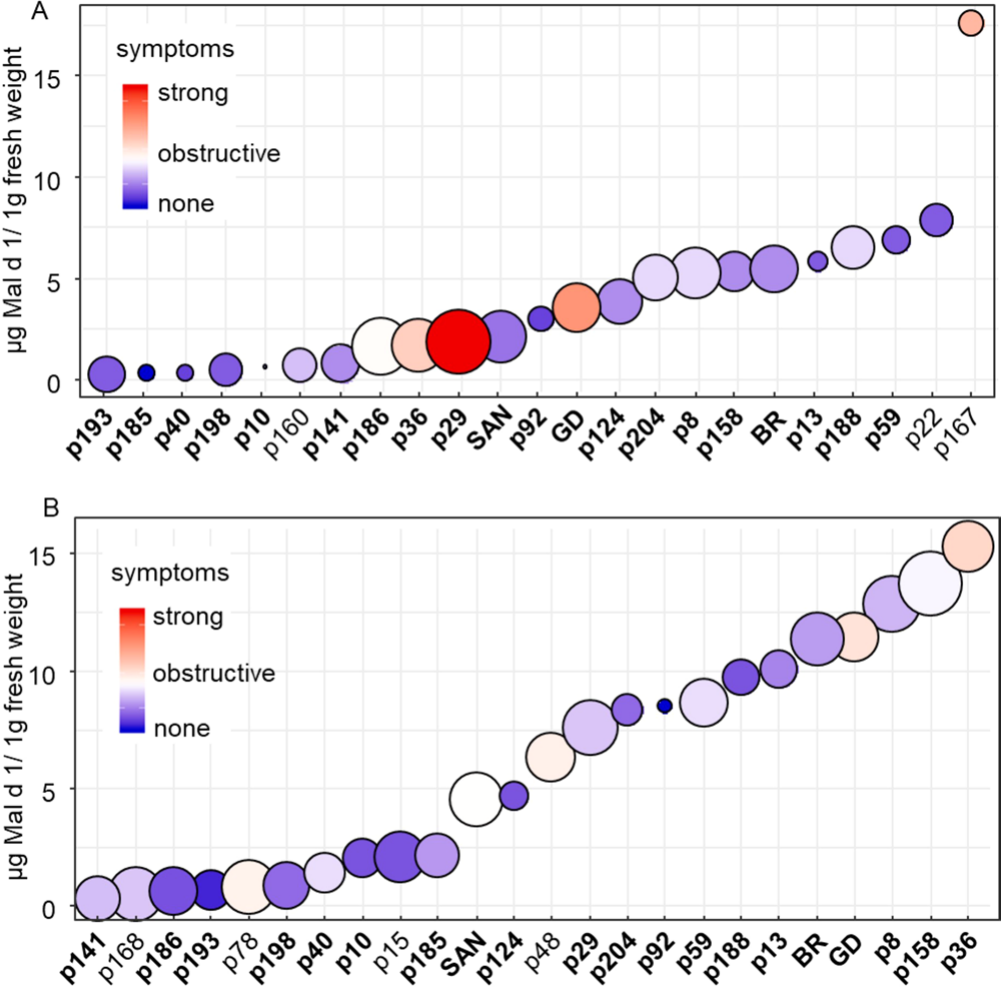
Results of the human study in comparison to the Mal d 1 concentration in µg/g fw of genotypes of the crossbreeding population and 3 standard cultivars (Santana (SAN), Golden Delicious (GD) and Braeburn (BR)) from harvest year 2017 (**A**) and harvest year 2018 (**B**) determined with ELISA-assay. The colour indicates the mean value of the tolerability of the apples. The size of the bubbles indicated the variance between the patients. The genotypes analysed in both years are highlighted in bold. The image was generated with RStudio 3.6.0(https://rstudio.com).

As a next step, a human study was conducted to investigate the tolerability of 20 selected genotypes for allergic patients (Fig. 2). Apples from two harvest years (2017 and 2018) were tested (Supplementary material S1). The genotypes were selected for their low Mal d 1 content, which was determined in 2016. Additional promising genotypes were included in 2018 (p 168, p78, p15, and p48), while genotypes with high Mal d 1 content in 2017 were excluded in 2018 (p22 and p167) and p160 did not yield sufficient fruit. The hypoallergenic cultivar Santana, allergenic Golden Delicious and Braeburn, as well as p167 (for 2017) with proven high allergen concentration were chosen for comparison. The Mal d 1 content of all genotypes and cultivars was determined in each harvest year before the provocation test was performed.

In 2017, most of the apple genotypes were well tolerated by allergic patients and provoked little or no symptoms (Fig. 2A, bluish color) however; specific genotypes did induce severe allergy symptoms. The best-tolerated genotype was p185, for which 10 out of 11 allergic person showed no or clinically irrelevant symptoms. In contrast, allergic patients who ate p36, p29, Golden Delicious or p167 reacted strongly with multiple symptoms, such as strongly swollen oral mucosa, lips or tongue, or other symptoms such as severe itching or watery eyes (Fig. 2 A, reddish color). All these symptoms were evaluated as not acceptable and disruptive. A comparison of the Mal d 1 concentration determined by ELISA assay and the results of the human study indicated that the Mal d 1 levels in apples do not correlate directly with the corresponding symptoms. Although cultivar Santana and genotype p29 had similar Mal d 1 levels, they provoked very different reactions in allergic patients. Golden Delicious is one of the cultivars with the highest allergenic potential but contained only a moderate content of Mal d 1 (Fig. 2A).

The large influence of environmental conditions on the Mal d 1 concentration and the tolerability was particularly evident after testing the Mal d 1 content in apples of the harvest year 2018. Most of the genotypes, such as p29, p204, p92, p158, p8 and the cultivars Golden Delicious and Braeburn had a significantly higher Mal d 1 concentration in the 2018 harvest year than in 2017. However, genotypes p193, p198, p141, p186, and p40 contained low Mal d 1 levels in both years and p124 accumulated a moderate concentration of 5 µg/g fw in 2017 and 2018. A correlation coefficient of 0.71 was calculated for the Mal d 1-content of the harvest years 2017 and 2018 using the *Spearman rank correlation*. In general, the apples from 2018 were better tolerated than the fruits from 2017 (Fig. 2B), although their Mal d 1 concentrations were usually higher. The strongest symptoms were observed after consumption of Golden Delicious and p36, but these symptoms were much less severe than in 2017.

### Polyphenol content

**Figure 3:**
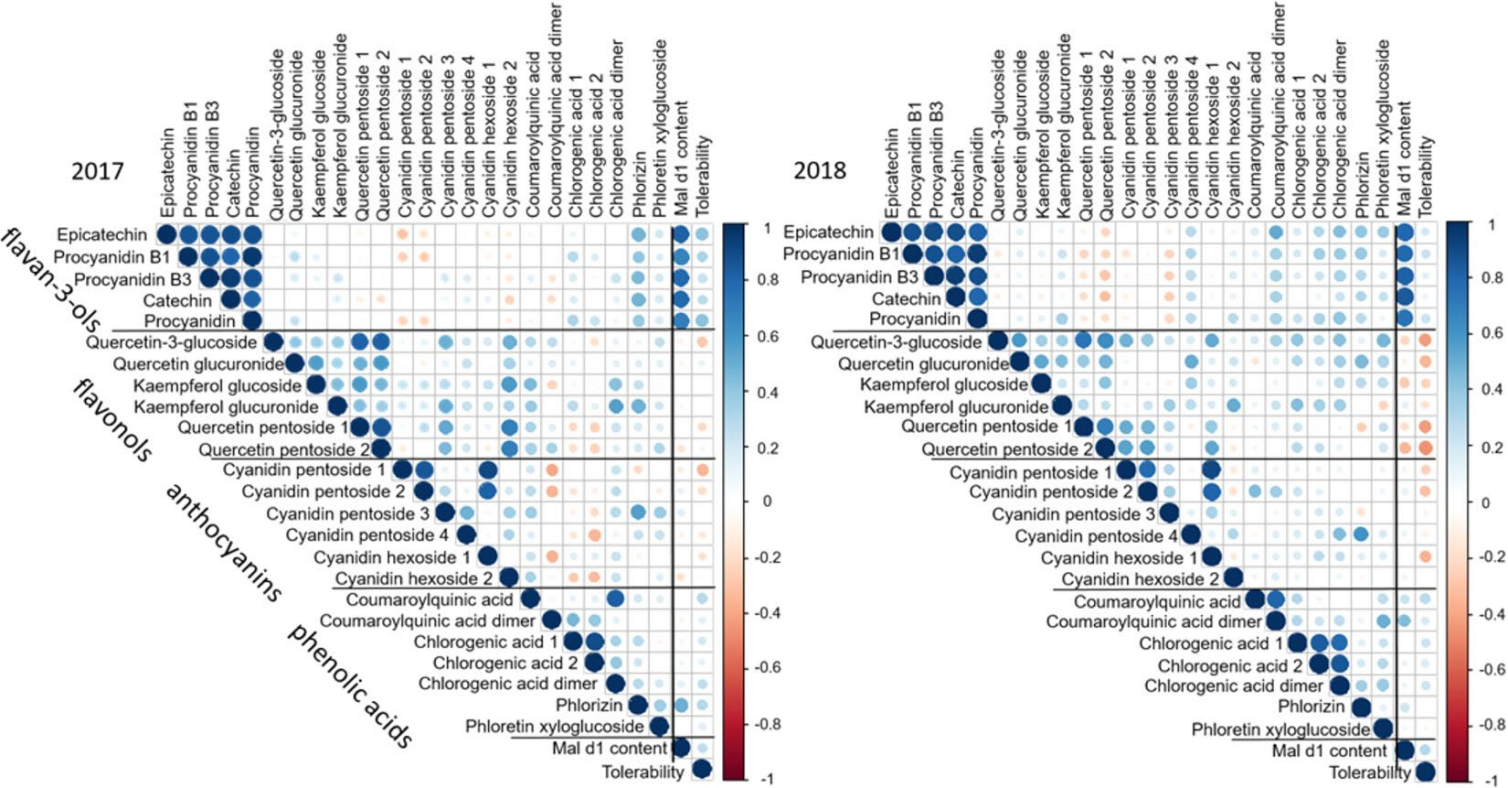
Correlations of polyphenols, allergen content and tolerability of apples harvested 2017 and 2018 calculated with *Spearman rank correlation-*test. The size of the bubbles indicates the value of the correlation coefficient, while blue bubbles indicate a positive correlation coefficient and red bubbles a negative correlation coefficient. The image was generated with RStudio 3.6.0 (https://rstudio.com).

**Figure 4:**
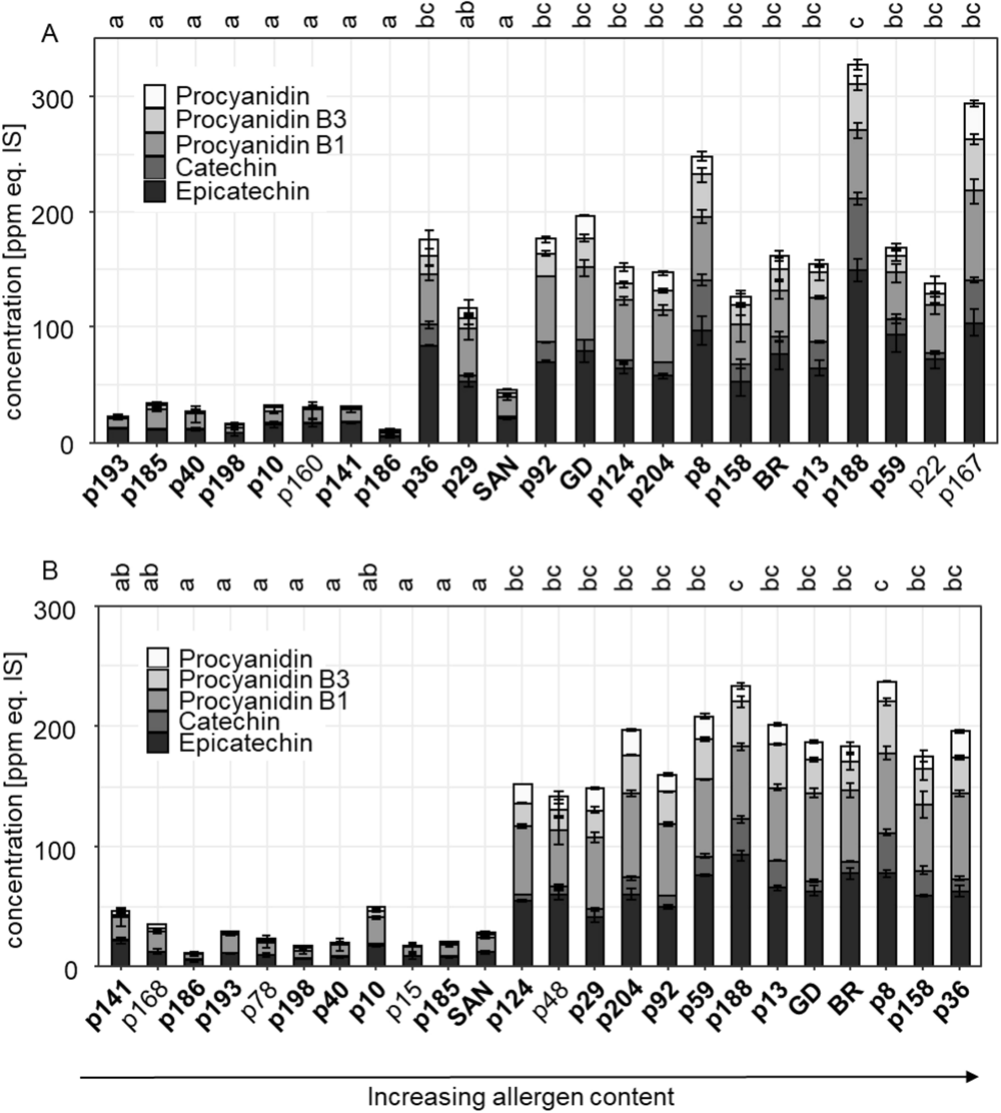
Relative concentration of flavan-3-ols in different apple genotypes of the crossbreeding populations and cultivars Santana (SAN), Golden Delicious (GD) and Braeburn (BR) from harvest year 2017 (**A**) and 2018 (**B**) in ppm equivalents of the internal standard (IS) biochanin A. The bars are ordered according to the increasing allergen content. The genotypes analysed in both years are highlighted in bold. The letters a-c indicate the significant differences at a significance level of 5% (*p* value ≤ 0.05) calculated with Dunn’s test. Different letters display a significant difference between the genotypes. The image was generated with RStudio 3.6.0 (https://rstudio.com).

For the genotypes whose Mal d 1 contents and tolerability were already known, polyphenols such as flavan-3-ols, flavonols, phenolic acids and anthocyanins were quantified by LC-MS/MS using an internal standard method. The levels of flavonols, anthocyanins, and phenolic acids showed genotype dependent differences but no correlation with the allergen content or tolerability was observed (Fig. 3). Strikingly, the flavan-3-ols catechin and epicatechin and the proanthocyanidins procyanidin B1, B3, and an unknown procyanidin showed a high positive correlation to the allergen content. The amount of these substances varied from 10 ppm equivalents of the internal standard (ppm eq. IS) for p 186 in 2017 to 326 ppm eq. IS for p 188 in 2017 (Fig. 4). The bars in Fig. 4 are arranged according to the increasing allergen content. The quantity ratios of individual flavan-3-ols are similar for the genotypes. Epicatechin was quantitatively the most important with 38% to 56% of total flavan-3-ols followed by procyanidin B1 with up to 50%. None of the genotypes showed a total flavan-3-ol concentration between 50 and 100 ppm eq. IS.

In 2017, a positive correlation between the allergen content and the total flavan-3-ol concentration was discernible (Figs. 3 and 4). Genotypes with a low allergen content of up to 1.5 µg/g fw (p186) showed a flavan-3-ol concentration of less than 35 ppm eq. IS. Genotypes with an allergen concentration of more than 3 µg/g fw (p92) had an at least fourfold higher flavan-3-ol content with more than 120 ppm eq. IS. In the middle range of the Mal d 1 concentration, p 36 (1.6 µg/g fw), p 29 (1.97 µg/g fw), and Santana (2.2 µg/g fw) showed similar allergen levels but the total flavan-3-ol contents were significantly different. Santana had a low flavan-3-ol concentration of 46 ppm eq. IS while the levels in the cultivars p36 and p29 were 178 and 116 ppm eq. IS, respectively. In the human study, Santana was well tolerated by allergic person but p36 and p29 provoked severe allergic symptoms. A correlation between allergen content and flavan-3-ol concentration was evident and was confirmed by *Spearman rank correlation* with a correlation coefficient of 0.76 (Fig. 3). Flavan-3-ol concentration and the tolerability of the genotypes showed no significant correlation (*Spearman rank correlation coefficient* 0.31).

The data obtained in 2018 confirmed the data of 2017. The genotypes containing less than 4.6 µg Mal d 1 per gram fw produced less than 50 ppm eq. IS of flavan-3-ols, and above a Mal d 1 concentration of 4.6 µg/g fw the genotypes accumulated more than 140 ppm eq. IS of flavan-3-ols. The correlation coefficient of the allergen content and the total amount of flavan-3-ols was 0.8 (Fig. 3). The flavan-3-ol content was less affected by the climactic and cultivation conditions than the allergen content as the comparison of the maximum values in both years showed. The proanthocyanidin concentration was strongly genotype-dependent. The genotypes with a high flavan-3-ol content, over 100 ppm eq. IS, showed this strong dependency in both years without exception. The correlation coefficient of the proanthocyanidin contents in two different harvest years was 0.89.

### Proteomic analysis of Mal d 1 via LC-MS/MS allows isoform detection

**Figure 5:**
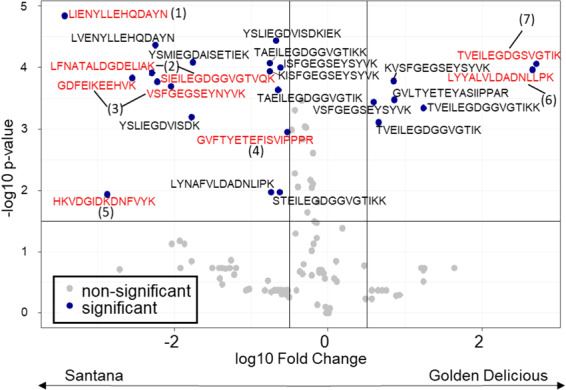
Vulcanoplot of peptides derived from Mal d 1 isoforms showing contrasting contents in apples of the cultivars Santana and Golden Delicious. The peptides with a positive log10 Fold Change (FC) were mainly present in Golden Delicious (GD), the peptides with a negative FC were mainly found in Santana. The peptides that are specific for single Mal d 1 isoforms or small groups are marked in red, more general peptides are black. Santana: Group of Mal d 1.03 proteins (**1**), CBL94177.1_Mal d 1.08 (**2**), group of Mal d 1.06 proteins (**3**), CBL94138.1_Mal d 1.10 (**4**), XP_008351173.1_Mal d 1-like protein (**5**) Golden Delicious: AAS00045.1_Mal d 1-like protein (**6**) and AAX20977.1_Mal d 1.06B or AAS00045.1_Mal d 1- like protein **(7)** (UniprotKB). Significance difference was calculated at significance level of 3% (*p* value ≤ 0.03). The image was generated with RStudio 3.6.0 (https://rstudio.com).

**Figure 6:**
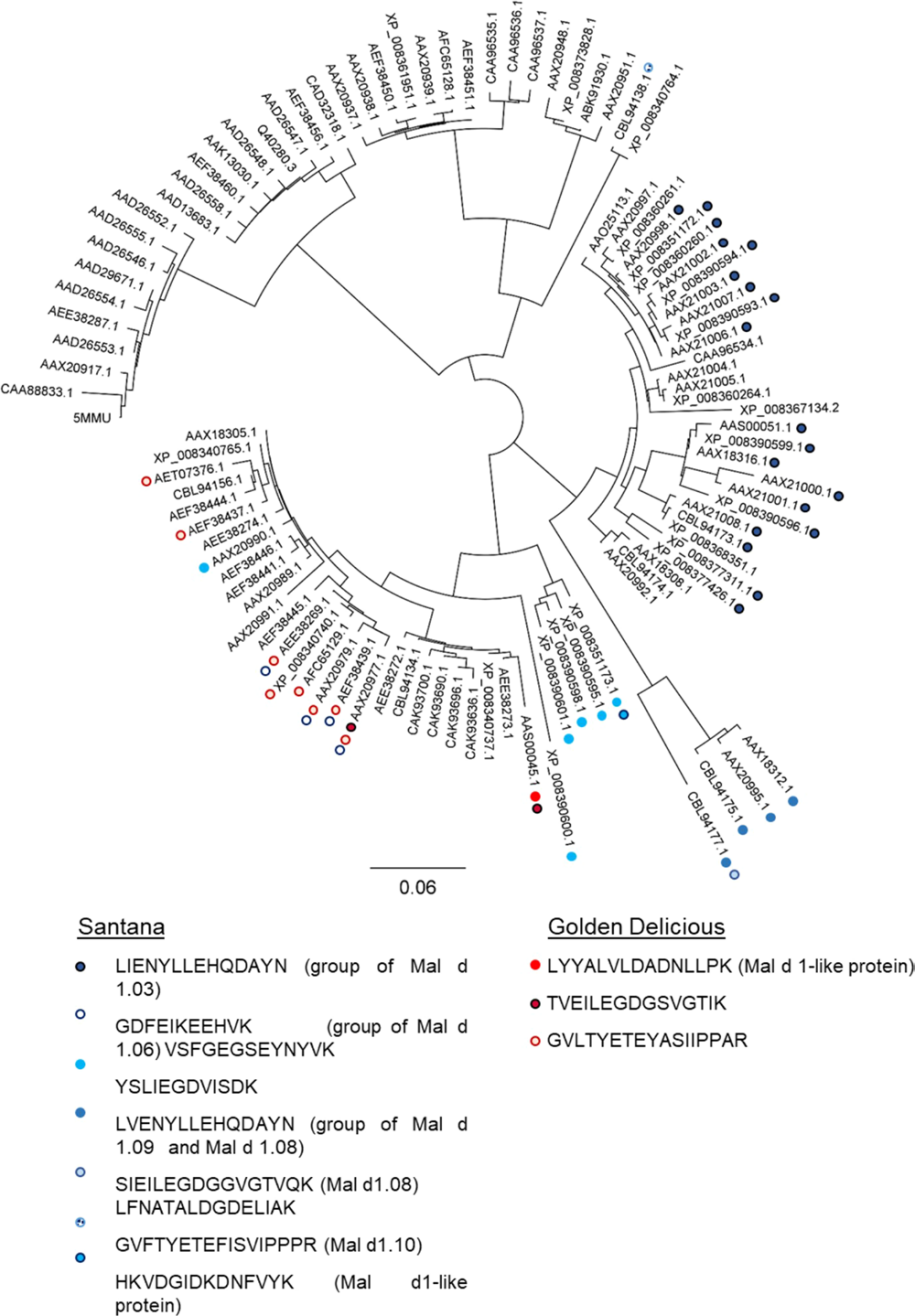
Phylogenetic tree of Mal d 1 isoforms (constructed with Geneious using the default values, Drummond *et al*. 2012). The specific peptides for Santana and Golden Delicious and their occurrence in the isoforms are marked. The image was generated with Geneious 5.6.7 (https://www.geneious.com).

**Figure 7:**
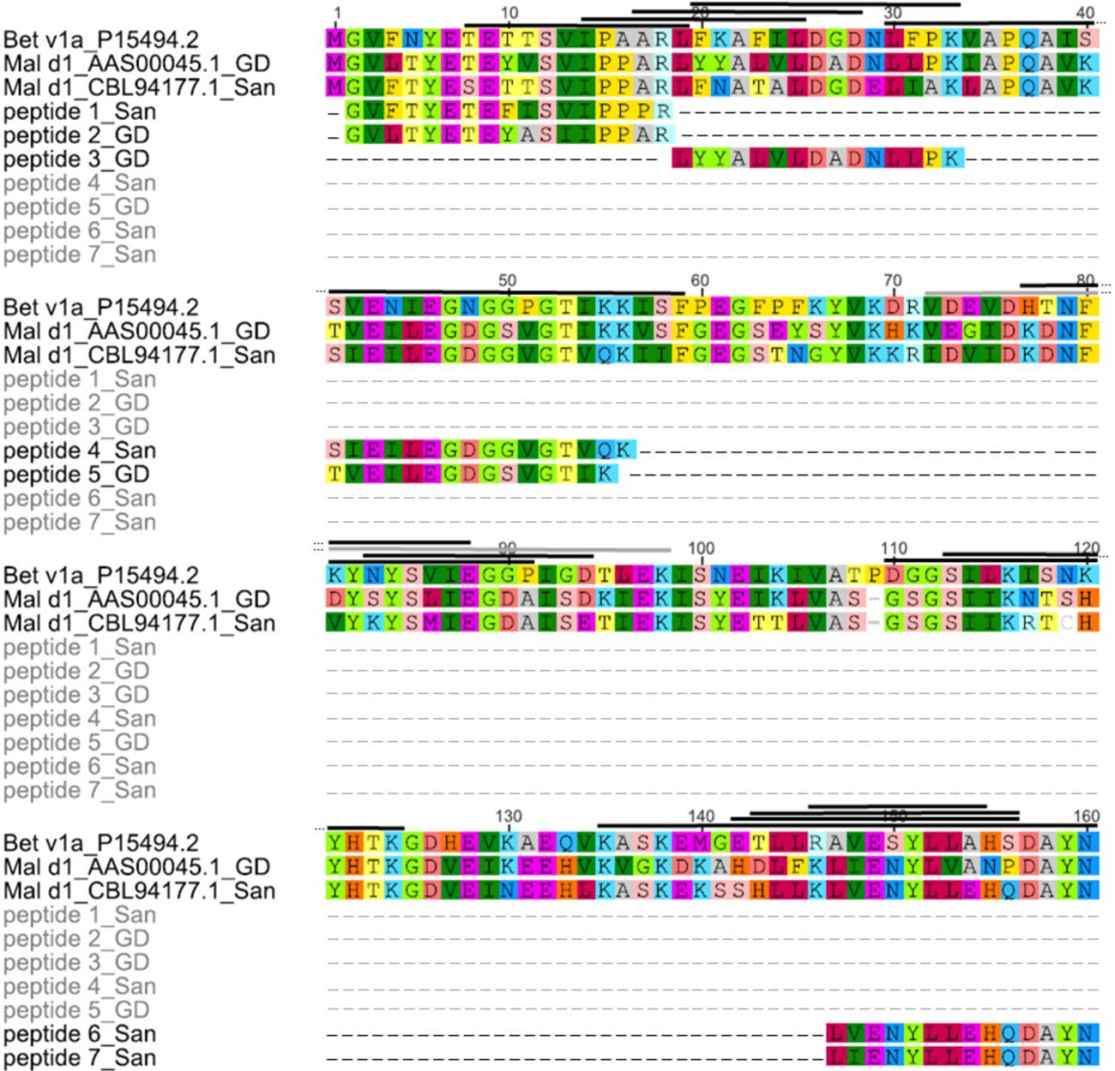
Mapping of selected peptides identified by LC-MS on the protein sequence of Bet v 1a and two specific Mal d 1 isoforms. The black bars above the protein sequence indicate IgE-epitopes of Bet v 1, identified and described in several publications (www.iedb.org Organism: *Betula verruscosa*; Antigen: Bet v 1). The image was generated with Geneious 5.6.7 (https://www.geneious.com).

Since Santana and Golden Delicious had similar Mal d 1 contents in 2017 but were tolerated differently by patients affected by allergy, we assumed that contrasting isoallergens were responsible for this observation. Therefore, the composition of Mal d 1 isoallergens in Santana and Golden Delicious apples were analysed by LC-MS/MS. The LC-MS/MS data were compared with a protein sequence database consisting of *M*. × *domestica* protein sequences and 113 known Mal d 1 isoforms and homologous sequences extracted from the NCBI database. In total, 117 tryptic peptides were assigned to Mal d 1 proteins in the samples (Supplementary material S2) and a two-sided t-test was used to find those peptides that were significantly differentially expressed in Santana versus Golden Delicious (Fig. 5). The peptides *LIENYLLEHQDAYN* (1) and *GDFEIKEEHVK* (3) were only found in protein extracts from Santana (Fig. 5). Due to the high structural similarity of the different isoallergens a clear assignment of the peptides to only one isoforms was usually not possible. However, the peptide *LIENYLLEHQDAYN* (1) that was highly abundant in Santana is located at the position 146–159 of the consensus sequence of Mal d 1 and could be assigned to a group of Mal d 1.03 isoforms (Figs. 6 and 7), which showed amino acid sequence identity between 89.4% and 99.4%. Similarly, peptides *GDFEIKEEHVK* and *VSFGEGSEYNYVK* (3) were assigned to the same isoform Mal d 1.06 (NCBI: AAX20977.1, AEE38269.1, AAX20979.1, AEF38439.1). This group of four possible isoforms differs in single amino acids. They have an amino acid sequence identity between 96.9% and 99.4%. An assignment to a single sequence was not possible. However, a few detected peptides in Santana were unique for only one Mal d 1 isoform. The peptide *GVFTYETEFISVIPPPR* (4) could be assigned to the isoform Mal d 1.10 (NCBI: CBL94138.1) as well as the peptide *HKVDGIDKDNFVYK* (5) that is unique for a Mal d 1-like protein (NCBI: XP_008351173.1). Two further detected peptides that can be found only in Santana *SIEILEGDGGVGTVQK* and *LFNATALDGDELIAK* (2) are both specific for one single isoform, Mal d 1.08 (NCBI: CBL94177.1). Some exclusive and specific peptides have also been identified in Golden Delicious. The peptide *LYYALVLDADNLLPK* (6) was unique for the isoform called Mal d 1-like protein (NCBI: AAS00045.1). The peptide *TVEILEDGDSVGTIK* (7) could be assigned to the same isoform but also to the Mal d 1.06B isoform (NCBI: AAX20977.1). Further peptides, exclusively present in one of the cultivars could be identified, but they are more general peptides that could not be precisely attributed to single isoforms. Some peptides and their occurrence in the different isoforms are displayed in Fig. 6. It appears that that the peptides, which are exclusively present in one apple genotype correlate well with phylogenetic similarity.

## Discussion

### Allergen content in different apple genotypes and their tolerability

As has already been observed with other homologous Bet v 1 proteins of the family of Rosaceae, the allergen content of apples is cultivar-dependent^35–38^. However, the variance of concentrations between different apple cultivars is higher than the variance between cultivars of other taxa from Rosaceae such as strawberry, *Fragaria* spp^35^. The Mal d 1 concentrations quantified in this study are within the range determined by others^5,14,39^. The cultivar dependency of the allergen content in two different harvest years (2017 and 2018) was confirmed by a correlation factor of 0.71 although the influence of cultivation techniques and environmental conditions was not taken into account. It should also be considered that the variability of the Mal d 1 concentration within a genotype, even within one apple fruit can be very high. Similarly, allergy to Mal d 1 is characterized by significant inter-patient variability^5,39^. In 2018, genotypes generally accumulated higher Mal d 1 concentrations than in 2017, indicating a climatic effect. The allergen content is also affected by additional factors, such as ripening stage and storage conditions^14,18,33^ that were kept identical in this study. In addition to the formation of Mal d 1 during fruit ripening^40^, this PR-10 protein, is also produced by abiotic or biotic stresses. Temperature, light, and water stress induced the expression of different *Mal d 1* genes^16^. In our study, Golden Delicious, which was classified as highly allergenic^5,14^, and the genotypes p158 and p36 showed a significant higher Mal d 1 content in 2018 than in the previous year. In contrast, cultivar Santana and genotypes p141, p193 and p198 did not differ much in the Mal d 1 concentration suggesting a more stable expression level. However, the human study showed a better tolerability of the apples harvested in 2018. The symptoms were not so severe and mostly clinically irrelevant. This leads to the hypothesis that the hot and dry summer of 2018 caused stressful conditions for the plants but did not increase the allergenic potential of the fruits. One reason might be the heat instability and thus loss of IgE antibody recognition of Mal d 1 proteins^41^. The specific polyclonal anti-Mal d 1 antibody used in this study was made for capturing native and denatured proteins by sensitizing the rabbit with denatured protein. Western blot experiments proved the recognition of the denatured Mal d 1 protein by the polyclonal antibody as well as homologous proteins of other fruit species. This means that the antibody used is also able to detect the different Mal d 1 isoforms in the apple fruit (Supplementary material S3). As shown also by others^39^, in this study a direct correlation was not observed between the Mal d 1 concentration and the corresponding symptoms, suggesting that the allergic reaction is not only influenced by the total Mal d 1 allergen content^30^. Different Mal d 1 isoforms may have different allergic potential, as has been shown for different Fra a 1 isoforms^62^. A reaction to other allergenic proteins in apples, such as Mal d 3 or the secondary allergens Mal d 2 and Mal d 4 may also possibly be the reason for the different tolerance, although Mal d 1 is described as the main allergen for apples in Central Europe. Furthermore, our and other results suggest that additional factors influence the tolerance of apples. In this context, the polyphenol content of apples seems to play a role, as confirmed by our LC-MS/MS analyses.

### Polyphenol analysis

The formation of Mal d 1 as a reaction of plants to different stress conditions is well known^42^ but also polyphenols^43^ such as flavan-3-ols, including catechin, epicatechin and proanthocyanidins play an important role in antimicrobial context^44^. It is assumed that polyphenols show anti-allergic potential through different mechanisms. One process is the lack of antibody recognition due to a changing tertiary structure of the protein. This can either be caused by polyphenols, which act as ligands for the hydrophobic cavity^45,46^, or caused by the enzyme polyphenol-oxidase (PPO). This enzyme catalyses the oxidation of polyphenols to quinones, that have antimicrobial activity^47^ and leads to enzymatic browning of fruits and vegetables. Due to structural similarity, PPO can also use the amino acid tyrosine as substrate. Oxidation of tyrosine in allergens led to the formation of covalent crosslinks within the proteins^48,49^ that resulted in a conformational change and a loss of antibody recognition. In a study with extracts from apple peels and cherry, the addition of catechin and polyphenol oxidase (PPO) significantly reduced the IgE binding to the allergens^50,51^. Others showed that the activity of PPO is more important than the content of total phenols to reduce the Mal d 1 level^52^.

The second mechanism for the anti-allergic effect of polyphenols is their effect on the mast cells and the prevention of histamine secretion^31,53,54^. Polyphenols of apples influenced the binding between IgE antibodies and the FCεRI receptor on the mast cells^55^. The reduced binding led to a lower amount of released histamine.

In this study, no correlation between total polyphenol and the allergen content was observed, but a strong dependence of flavan-3-ol and allergen concentration was revealed. Most previous studies reported a positive correlation between the tolerability of apples and the polyphenol content. Higher concentrations of epicatechin, as well as caffeic acid and chlorogenic acid resulted in a better tolerability of the apples^30^ and the addition of catechin to an apple peel extract reduced the IgE binding capacity^50^. It was concluded that high concentrations of flavan-3-ols could decrease, like other polyphenols, the allergenic potential of the protein and decrease the symptoms by changing the conformational epitopes or preventing degranulation of the mast cells^31^. This could explain the good tolerability of some genotypes with a high allergen content in the human study.

In addition, the binding of flavan-3-ols to Mal d 1 homologous proteins in *Fragaria* spp. was reported. It was shown by crystal structure analysis that (+)-catechin acted as ligand Fra a 1 proteins^45^. The affinity of the protein to (+)-catechin was isoform-dependent^56^. The isoform dependence of ligand binding was also observed with Bet v 1^57^. The binding of flavan-3-ols in the hydrophobic cavity of Mal d 1 has not yet been observed, but it could lead to a coexistence of flavan-3-ols and Mal d 1 and would explain the positive correlation of the concentrations similar to the binding of quercetin sophoroside to Bet v 1^58^.

### Isoforms of Mal d 1

In addition to the ligand specificity of the isoallergen forms, a difference in the RNAse activity and cytokinin binding activity was observed for isoallergens^59^. This led to the assumption that different isoforms have different functions in the defence mechanisms in plants and show different allergenic potential despite their high structural similarity^59,60^. The different allergenic potential of various isoforms has already been described^61–63^.

*Mal d 1* forms a multigene family and 31 genes have been described for Golden Delicious^15^. Seven of them contain introns^64^. The expression of these genes varies between the different genotypes and plant tissues, like leaves, pollen and fruits^16,65^. The *Mal d 1* gene expression is also depending on the ripening stage^66^, the storage time^18^ and different stress conditions like pathogen or water-stress^16,67^. LC-MS based proteome analyses after tryptic digestion of fruits and detection of allergens have already been performed^68,69^. Analysis of birch pollen proteins revealed 43 peptides of 11 different Bet v 1 isoforms^70^, which were later confirmed for additional genotypes^71^ but a comprehensive targeted analysis of Mal d 1 proteins has not been performed until now. The high structural similarity of the Mal d 1 isoforms makes the analysis considerably more difficult because some isoforms differ in only one amino acid and not all unique specific peptides are detectable due to the different ionizability of the peptides. Nevertheless, proteomic analysis of isoforms has a major advantage over PCR-based gene expression analysis since gene expression data cannot reliably quantify the clinically relevant isoforms^72^. A wide variety of orthologous isoforms, encoded by only a single Bet v 1 gene of the gene family was found in different birch genotypes^73^. In apples up to six different protein variants (for *Mal d 1.06 C*) were characterised in different genotypes, while a maximum of two variants per genotype are possible^64^. Coupled mass spectrometry approaches create new opportunities to identify the isoforms that are relevant for allergic patients. The analysed cultivars Santana and Golden Delicious, which showed similar total allergen concentrations but different allergenic potential in 2017 (Fig. 2A), exhibited large differences in their Mal d 1 isoform composition. Many peptides identified by LC-MS were unique to one of these cultivars, meaning that they were found in only one of two. Therefore, the contrasting isoallergens detected in the cultivars could explain the different allergenic potentials.

Analysis of different peptides derived from Mal d 1 led to the identification of epitopes that affect T-cells stimulation^74,75^. Equivalent to the BV16 epitope of Bet v 1^76^, which contains the glycine-rich-loop between the helices β2 and β3 and other conserved amino acids, Mal d 1 shows a similar motif with 13 identical amino acids out of a total of 16^61,77^. Mutation of single amino acids improved IgE recognition, underlining the importance of single amino acids^77^. Our analysis revealed some unique peptides, including those with the highly conserved glycine-rich-loop found also in the BV16 epitope, such as *TVEILEGDGSVGTIK* (Golden Delicious; aa 41–55) and *SIEILEGDGGVGTVQK* (Santana; aa 41–56; Fig. 7). The position of most of the detected peptides could be linked to the region of published epitopes (www.iedb.org) such as *LIENYLLEHQDAYN* (aa 147–160) and *LVENYLLEHQDAYN* (Santana; aa 147–160). Positions 142–156 and 143–153 are presumed epitopes (www.iedb.org). Similarly, peptides *GVLTYETEYASIIPPAR* (Golden Delicious) and *GVFTYETEFISVIPPPR* (Santana) are located at position 2–18, which overlap with an assumed epitope at position 8–19. It is striking that the peptide *LYYYALVLDADNLLPK*, which was identified only in Golden Delicious (aa 19–33) after tryptic digestion, falls into a region of the allergen where several epitopes have been identified. Therefore, we assume this peptide could be a good candidate to distinguish fruits with high and low allergenic potential, taking into account, that differences in IgE recognition of different patients to single peptides and epitopes have been shown^74^. Further experiments are needed to verify this hypothesis.

## Conclusion

**Figure 8:**
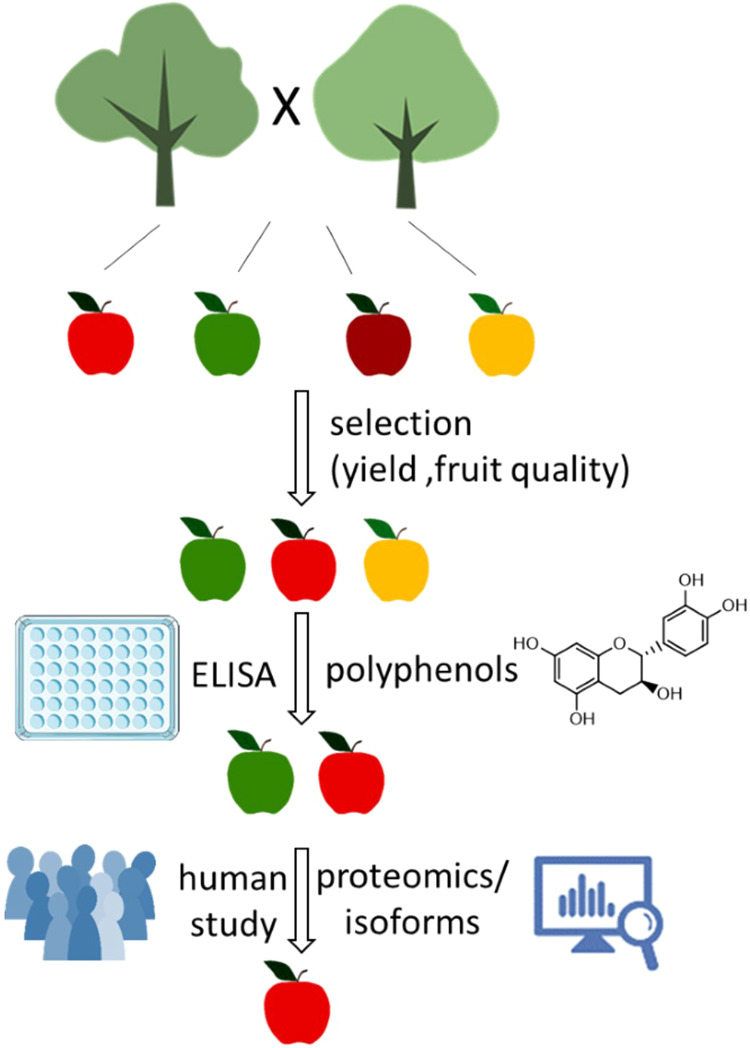
Tiered approach for the identification of Mal d 1 reduced, well tolerated apple genotypes. The image was generated with PowerPoint (https://www.microsoft.com).

The breeding of hypoallergenic apples requires the identification of genotypes with low allergen content that are stable over harvest years and are not affected by environmental changes. The most important criterion, however, is good tolerability, as allergy sufferers must not experience any symptoms. Therefore, we performed a tiered approach, combined ELISA-based Mal d 1 quantification with human provocation studies, and demonstrated that apples with a low allergen content are not necessarily well tolerated (Fig. 8). The flavan-3-ol concentration and the composition of Mal d 1 isoforms were identified as additional factors putatively modulating the allergenic potential of apple genotypes. With the tiered approach presented, we found in breeding lines, due to the great natural diversity, candidates (p141, p185, p198, and p10) that meet the above-described requirements. For two consecutive years, these genotypes produced less Mal d 1 and were better tolerated than the allergy-friendly Santana.

## Materials and Methods

### Chemicals and reagents

The chemicals were purchased from Carl Roth (Karlsruhe, Germany) unless otherwise noted.

### Plant materials

The segregating populations were produced and were cultivated by the University of Applied Sciences Osnabrück in cooperation with the privately financed organization “Züchtungsinitiative Niederelbe GmbH & Co. KG”. The breeding lines were obtained from manifold combinations in crossing, using established cultivars as well as preselected breeding lines as parents, hence reflecting a profile of high-quality apples. As parental cultivars Honeycrisp, Braeburn, Gala, Dalinbel, Santana, Pinova, Gloster, Topaz, Golden Delicious, Rubinette, Elstar, Delbarestivale, Retina, Rubens, Fuji and Nicoter were used. A correlation between the Mal d 1 content of parental cultivars and progenies could not be observed. The examined apple clones and varieties were harvested in the years 2016, 2017 and 2018 between mid-August and mid-October, depending on the picking ripeness. The apples were harvested from five trees per genotype. All trees were cultivated on the experimental plots of the Osnabrück University of Applied Sciences and the “Züchtungsinitiative Niederelbe” in Northern Germany. Immediately after harvesting, one quarter of each of three ripe apples was cut into pieces and rapidly frozen at −20 °C. The frozen pieces, containing flesh and skin, were ground with liquid nitrogen to a fine powder and stored at −80 °C until ELISA and LC-MS/MS analysis. Since the allergy suffers in this study could test a maximum of two apples per day and the provocation test could not be performed in the immediate vicinity of the test field, it was not possible to test the apple genotypes exactly when the other apples were frozen for the ELISA measurement. Therefore for the human provocation study, at least 40 apples of each genotype were stored under ultra-low-oxygen-condition (2.5 °C; controlled atmosphere, 1.5% O_2_; 1% CO_2_) for 31 to 167 days, depending on the maturity for consumption until dispatch to the Allergy-Centre-Charité Hospital, Berlin, Germany. The timing of the analyses in the different years differed by only 20 days.

### Production and purification of recombinant Mal d 1.02 in *Escherichia coli*

The recombinant Mal d 1 (rMal d 1.02; accession number Q8L6K9), as a fusion protein with a C-terminal polyhistidin-tag, was heterologously expressed and produced after transformation of *Escherichia coli* BL21 (DE3) pLysS strain with the pQE70 vector carrying *mal d 1*-gene. The cells were cultivated in LB medium containing 100 µg/mL ampicillin and 34 µg/mL chloramphenicol at 37 °C until an optical density OD_600_ of 0.6 was reached. The protein production was induced with 100 µg/mL isopropyl β-D-1-thiogalactopyranoside (IPTG) and the culture was incubated for 20 h at 18 °C. After cell harvest by centrifugation (20 min, 5,292 *g*, 4 °C), the pellets were stored at −80 °C. The cell pellet was resolved in Binding Buffer (50 mM Tris, 220 mM NaCl, 10 mM imidazole, pH 7.5) containing 0.5 mM phenylmethylsulfonyl fluoride (PMSF) (Sigma-Aldrich, Taufkirchen, Germany), 1 mM MgCl_2_ (New England Biolabs, Frankfurt, Germany), 1 µL DNAse and 0.5 mg/mL lysozyme. The cells were disrupted by ultrasonication. After centrifugation (30 min, 4 °C, 21,191 *g*) the insoluble inclusion bodies were dissolved in denaturation buffer (6 M urea, 20 mM Tris-HCl, 220 mM NaCl, 5 mM imidazole, pH 8) overnight at 4 °C and centrifuged (1 h, 21,191 *g*, 4 °C). The supernatant was dialyzed (ZelluTrans, 3.5 kDa MWCO) against refolding buffer (10 mM NaH_2_PO_4_, 10 mM Na_2_HPO_4_, 500 mM NaCl, 20 mM imidazole) for 20 h. The solution was centrifuged (45 min, 21,191 *g*, 4 °C) and the supernatant was incubated with Profinity Immobilized Metal Ion Affinity Chromatography (IMAC) His-tag resin (Bio-Rad Laboratories, Feldkirchen, Germany) for 2 h at 4 °C. After three washing steps with each 10 mL binding buffer, the rMal d 1 protein was eluted 5 times with 300-µL elution buffer (50 mM Tris, 220 mM NaCl, 250 mM imidazole, pH 7.5). The elution fractions were pooled, dialyzed against sodium carbonate buffer (10 mM Na_2_CO_3_, 10 mM NaHCO_3_, pH 9; Fluka, Steinheim, Germany) overnight at 4 °C, lyophilized, and stored at −20 °C. Before use, the dried protein was resolved in bidestilled water containing 1 mM cysteine. The purity was verified with SDS-PAGE. One mg of rMal d 1 was sent to Davids Biotechnology GmbH, Regensburg, Germany for the production of polyclonal antibodies. ELISA and Western blot analyses showed that the polyclonal antibody used could detect denatured Mal d 1 and the denatured homologous proteins Bet v 1 from birch, Pru d 1 from plums and Fra a 1 from strawberry (Supplementary material S3). The sequence similarity of the isoforms of Mal d 1 is higher than the similarity of the homologous proteins from different species. The antibody used is therefore suitable for a general determination of different Mal d 1 isoforms.

### Mal d 1 extraction from apple

For protein extraction, 1 g of the powdered apple sample was homogenized with 3 mL extraction buffer containing 6 M urea, 20 mM Tris-HCl, 220 mM NaCl and 5 mM imidazole, pH 8 and was incubated for 4 h at 4 °C on a rotation shaker. After centrifugation (30 min 5,525 *g*, 4 °C) the supernatant was dialysed (ZelluTrans, 3.5 kDa MWCO) against sodium carbonate buffer (pH 9) at 4 °C overnight. Precipitates were removed by centrifugation and the supernatant was used directly for enzyme linked immunosorbent assay (ELISA).

### Indirect competitive ELISA

A 96-well microtiter plate (ImmunoGrade, Brand, Wertheim, Germany) was coated with 100 µL 0.5 µg/mL rMal d 1 in PBS buffer (10 mM NaH_2_PO_4_, 10 mM Na_2_HPO_4_, 150 mM NaCl, pH 7.4) overnight at 4 °C. One hundred µL PBS served as blank. After three washing steps each with 300 µL PBS containing 0.5% Tween-20 (PBS-T), the free binding sites of the wells were blocked with 1% Bovine Serum Albumin (BSA) in PBS for 1 h at room temperature. The plate was washed again with PBS-T three times. For the standard curve, seven different concentrations in a range of 0.0002–75 µg/mL of rMal d 1 in PBS-T were prepared (Supplementary material S4). Fifty µL of the standard curve solutions or apple extract were added to the plate, at least in triplicates. The apple extracts were used undiluted and diluted 1:2 with PBS-T. To each well 50 µL of the primary polyclonal antibody (Davids Biotechnology GmbH, Regensburg, Germany) in PBS-T was added (0.75 µg/mL). After an incubation time of 1 h, the plate was washed four times with PBS-T. One hundred µL of the secondary antibody (goat-anti-rabbit-HRP; Carl Roth, Karlsruhe, Germany) in PBS-T was added to each well and the plate was incubated for 1 h at room temperature. After five washing steps the plate was incubated with 100 µL 3,3′,5,5′-tetramethylbenzidine (TMB) (Thermo Fisher Scientific, Waltham, USA) for 3 min until the substrate turned blue. The enzymatic reaction was stopped with 100 µL 2 M sulphuric acid and the absorption at 450 nm and 620 nm was measured with CLARIOstar (BMG Labtech, Ortenberg, Germany).

### Human study

The human provocation study was performed at the Comprehensive Allergy Centre at the Charité Hospital in Berlin based on a positive vote from the Charité ethic commission (No. EA/195/15). The Charité ethic committee approved all experimental protocols. All methods were carried out in accordance with the relevant guidelines and regulations. Study participants gave their informed consent to participate in the study. The study was attended by 13 adult non-smoking (4 males and 9 females) between 22 and 67 years of age (median 52 years). They all had a positive anamnesis on allergic rhinitis due to birch pollen since more than 2 years and a positive skin prick test with >3 mm oedema, and had a proven allergy to apples. Birch pollen allergy suffers also cross-react with pollen from hazel, alder or other tree pollen. A minority of the patients (5 | 13) were also positive for grass pollen in the skin prick test and two of them showed a skin reaction after cat dander contact. No patient took any anti-allergic or antihistaminic medication. The test persons consumed 100 g fresh apple (flesh and skin), described, and evaluated the appearing symptoms after 20 to 30 min. The following clinical symptoms were recorded; itching or swelling of the lips, tingling in the mouth, swelling of the tongue, swelling of the oral mucosa, and other symptoms on a scale from 0 to 3 (0 = complete absence of symptoms, 1 = symptom present, minimal attention to the symptom, easy to tolerate, 2 = symptoms are perceived, obstructive but tolerable, 3= symptoms are difficult to tolerate and disturb daily life). When the symptoms decreased to scale 0 or 1, the patients tested the next apple genotype. The test persons tried a maximum of two apples per day. The study was conducted outside the birch pollen season.

### LC-MS/MS polyphenols

The extraction of the polyphenols was performed in triplicates according to Ring *et al*^78^. Five hundred mg frozen apple powder was extracted four times each with 500 µL methanol and the supernatants were combined. As an internal standard, the isoflavone biochanin A was added (0.2 mg/mL). The solvent was evaporated with a vacuum concentrator and remaining water was removed by freeze drying (1 h). The pellet was resolved in 35 µL water and used for liquid chromatography coupled to mass spectrometry (LC-MS/MS).

### Mal d 1 isoform detection by LC-MS/MS

For protein extraction, 1 g frozen apple powder was incubated with 3 mL of solubilisation buffer (pH 8) for 3 h at 4 °C. After centrifugation (20 min, 5,525 *g*, 4 °C), the proteins of the supernatant were precipitated with fourfold the amount of ice cold acetone overnight at −20 °C. The pellet was washed five times with each 1.5 mL of −90 °C ice-cold acetone and then dried at 35 °C on a thermoblock. In-gel tryptic digestion was performed according to a standard procedure^79^. Briefly, the dried pellets were resuspended in 100 µL Laemmli-buffer (Bio-Rad Laboratories, München, Germany) and were heated for 5 min at 95 °C. Disulfid bonds were reduced with 50 mM DTT at 70 °C for 10 min and cysteine residues were alkylated with 55 mM chloroacetamide (CAA) at RT for 30 min in the dark. The samples (30 µl) were run on a NuPAGE 4–12% Bis-Tris Protein Gel (Thermo Fisher Scientific, Waltham, USA) for 5 min (200 V, 500 mA, 1x MOPS buffer). Subsequently, the still not size-separated single protein band per sample was cut out of the gel and digested overnight with trypsin (Trypsin Gold, Promega, Fitchburg, USA). LC-MS/MS measurements of the tryptic peptides were performed on an Ultimate 3000 RSLCnano system coupled to a Q-Exactive HF-X mass spectrometer (Thermo Fisher Scientific, Waltham, USA). For each analysis, 5 μL of the solution containing the tryptic peptides was delivered to a trap column (ReproSil-pur C18-AQ, 5 μm, Dr. Maisch, 20 mm × 75 μm, self-packed) at a flow rate of 5 μL/min in 100% solvent A (0.1% formic acid in HPLC grade water). After 10 min of loading, peptides were transferred to an analytical column (ReproSil Gold C18-AQ, 3 μm, Dr. Maisch, 450 mm × 75 μm, self-packed) and separated using a 50 min gradient from 4% to 32% of solvent B (0.1% formic acid in acetonitrile and 5% (v/v) DMSO) at 300 nL/min flow rate. Both nanoLC solvents contained 5% (v/v) DMSO. The Q-Exactive HF-X mass spectrometer was operated in data dependent acquisition and positive ionization mode. MS1 spectra (360–1300 *m/z*) were recorded at a resolution of 60,000 using an automatic gain control (AGC) target value of 3e^6^ and maximum injection time (maxIT) of 45 msec. After peptide fragmentation using higher energy collision induced dissociation (HCD), MS2 spectra of up to 18 precursor peptides were acquired at a resolution of 15,000 with an automatic gain control (AGC) target value of 1e^5^ and maximum injection time (maxIT) of 25 msec. The precursor isolation window width was set to 1.3 *m/z* and normalized collision energy to 26%. Dynamic exclusion was enabled with 25 sec exclusion time (mass tolerance + /−10 ppm). Peptide identification and quantification was performed using MaxQuant (version 1.5.3.30) with its built-in search engine Andromeda^80,81^. MS2 spectra were searched against an apple (*Malus* × *domestica*) proteome fasta file downloaded from NCBI (download 11.01.2019, total protein entries 119,379). The fasta file was filtered for proteins associated to *Malus domestica* only and duplicated sequences removed. Furthermore, 100 Mal d 1 isoform sequences derived from Blast.NCBI (https://blast.ncbi.nlm.nih.gov/Blast.cgi) were added and again duplicates removed. In total the fasta file contained 52,099 apple protein sequences, entailing 113 isoform and variant sequences of Mal d 1. This file was further supplemented with common contaminants (built-in option in MaxQuant). For the MaxQuant analysis, carbamidomethylated cysteine was set as fixed modification and oxidation of methionine and N-terminal protein acetylation as variable modifications. Trypsin/P was specified as proteolytic enzyme. Precursor tolerance was set to 4.5 ppm, and fragment ion tolerance to 20 ppm. Results were adjusted to 1% false discovery rate (FDR) on peptide spectrum match (PSM) level employing a target-decoy approach using reversed protein sequences. The minimal peptide length was defined as seven amino acids; the “match-between-run” function was not enabled.

### Data analysis

The peptides identified as contaminants and reverse sequences were excluded from the downstream analysis. Next, the peptide intensities were logarithm transformed (base 10), then the overall intensities of each sample were shifted so that all samples had the same median intensities as the grand median. Six, a constant close to the lowest detected values in this experiment, replaced missing values. This is rationalized by the fact that low abundant proteins are more frequently resulting in missing values in the MS-based proteomics. To find Mal d 1 peptides (all other peptides were excluded at this step) that are differentially abundant in Santana and Golden Delicious, t-test was used. The resulted p-values from t-tests were corrected using the Benjamini and Hochberg method^82^ to control the False Discovery Rate (FDR) of differential expression.

## Supplementary information

Supplementary information.

Supplementary information 2.

Supplementary information 3.
