# Supplementary material for: Tiered approach for the identification of Mal d 1 reduced, well tolerated apple genotypes
Source: Sci Rep. 2020 Jun 4;10:9144. doi: 10.1038/s41598-020-66051-4 (PMC7272412; doi:10.1038/s41598-020-66051-4)
Supplement: Supplementary file 3 — Supplementary information 3. [file 41598_2020_66051_MOESM3_ESM.pdf]

Supplementary Information

Tiered approach for the identification of Mal d 1 reduced, well tolerated apple genotypes

Emilia Romer[1], Soraya Chebib[1], Karl-Christian Bergmann[2], Katrin Plate[3], Sylvia Becker[4], Christina Ludwig[5], Chen Meng[5], Thilo Fischer[6] , Werner Dierend[3], Wilfried Schwab[1]

[1]Biotechnology of Natural Products, Technical University Munich, Liesel-Beckmann-Str. 1, 85354 Freising, Germany

[2]Comprehensive Allergy Center Charité, Charité Hospital Berlin, Charitéplatz 1, 10117 Berlin Germany

[3]Faculty of Agricultural Science and Landscape Architecture, Fruit Science, University of Applied Sciences Osnabrück, Oldenburger Landstr. 24, 49090 Osnabrück, Germany

[4]European Center of Allergy Research Foundation, Robert-Koch-Platz 7, 10117 Berlin, Germany

[5]Bavarian Center for Biomolecular Mass Spectrometry (BayBioMS), Technical University Munich, Gregor-Mendel-Straße 4, 85354 Freising, Germany

[6]4GENE, Lise-Meitner-Str. 30, 85354 Freising, Germany

Supplementary material S3. Western blot analysis

Supplementary material S4. Standard curve and raw data example

Supplementary material S3.

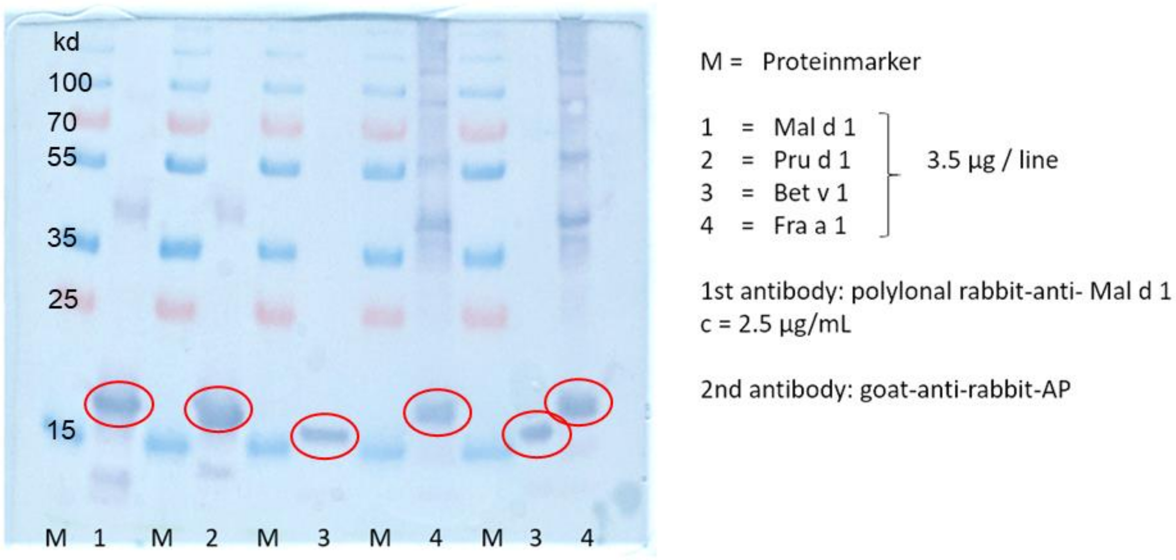

Western blot analysis of Mal d 1.02 (Q8L6K9), Pru d 1 (B5KVN9), Bet v 1 a (P15494), and Fra 1.02 (QET923) using the antibody raised against Mal d 1. Experimental details [35]. Prestained marker proteins were used.

Supplementary material S4.

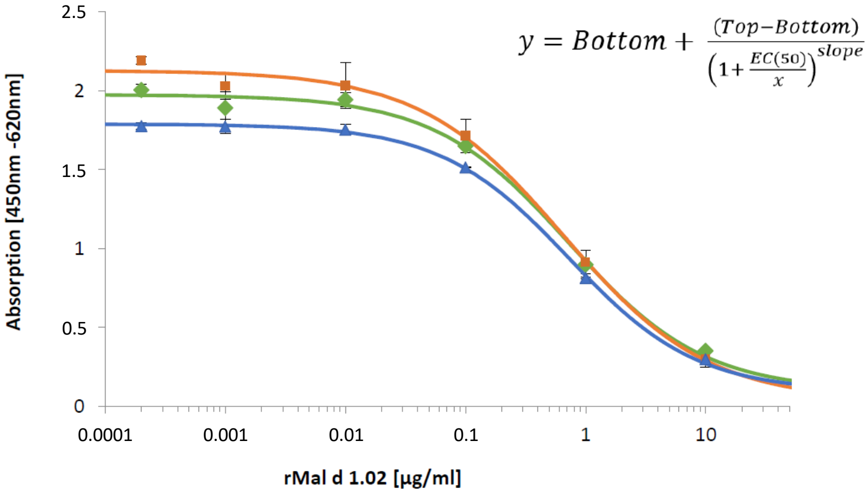

Standard curves of rMal d 1 calculated with the 4-parameter fit. On each ELISA-plate, a standard curve of serial diluted rMal d 1.02 in triplicates were performed to adjust inter-assay variations.

ELISA - Raw data of three exemplary apple genotypes with calculated Mal d 1 concentrations. Samples were used undiluted and diluted 1:2 in PBS-buffer to fit the linear range of the standard curve. For more details, see Materials and Methods. The prefix p indicates the genotypes of the crossbreeding population. Each line represents one measurement.

Sample	Raw data (OD_{450nm})	Raw data (OD_{620nm})	Absorption (blank corrected) (450 nm – 620 nm)	4-Parameter fit incl. dilution factor (µg/mL) = concentration in extract	Concentration Mal d 1 (µg/g FW) = concentration in apples
blank	0.1	0.029			
blank	0.084	0.03			
p 124	1.068	0.03	0.973	1.254	3.599
p 124	1.103	0.029	1.009	1.139	3.269
p 124	0.988	0.03	0.893	1.557	4.469
p 124	1.07	0.031	0.974	1.249	3.657
p 124	0.977	0.029	0.882	1.603	4.693
p 124	1.074	0.029	0.98	1.231	3.605
p 124 1:2	1.376	0.029	1.281	1.073	3.080
p 124 1:2	1.209	0.029	1.114	1.713	4.917
p 124 1:2	1.233	0.029	1.139	1.602	4.599
p 124 1:2	1.293	0.03	1.198	1.361	3.985
p 124 1:2	1.225	0.03	1.13	1.639	4.799
p 124 1:2	1.313	0.029	1.219	1.282	3.754
blank	0.069	0.03			
blank	0.064	0.029			
p 6	1.798	0.03	1.731	0.124	0.370
p 6	1.922	0.029	1.856	0.044	0.131
p 6	1.832	0.034	1.761	0.102	0.304
p 6	1.794	0.03	1.727	0.127	0.381
p 6	1.839	0.03	1.772	0.095	0.285
p 6	1.777	0.029	1.711	0.14	0.420
p 6 1:2	1.95	0.029	1.883	0.062	0.185
p 6 1:2	1.936	0.029	1.87	0.075	0.224
p 6 1:2	1.9	0.03	1.833	0.114	0.340
p 6 1:2	1.931	0.031	1.863	0.082	0.246
p 6 1:2	1.938	0.031	1.87	0.075	0.225
p 6 1:2	1.944	0.029	1.878	0.067	0.201
blank	0.102	0.032			
blank	0.099	0.029			
p 31	0.763	0.029	0.663	4.231	12.574
p 31	0.82	0.029	0.721	3.544	10.532
p 31	0.712	0.029	0.613	4.977	14.790
p 31	0.71	0.03	0.609	5.038	14.741
p 31	0.76	0.029	0.661	4.266	12.482
p 31	0.737	0.029	0.638	4.584	13.413
p 31 1:2	0.958	0.03	0.859	4.778	14.199
p 31 1:2	1.061	0.029	0.962	3.613	10.737
p 31 1:2	1.031	0.031	0.93	3.932	11.685
p 31 1:2	0.889	0.03	0.789	5.809	16.997
p 31 1:2	0.999	0.029	0.9	4.272	12.500
p 31 1:2	0.933	0.03	0.834	5.122	14.987
